# Inflammatory blood count ratios discriminate disease activity and remain persistently elevated during glucocorticoid-free remission in ANCA-associated vasculitis

**DOI:** 10.1007/s10238-026-02236-x

**Published:** 2026-06-22

**Authors:** Federica Davanzo, Luca Iorio, Michela Pelloso, Veronica Davanzo, Roberta Prevedello, Andrea Padoan, Chiara Baggio, Francesca Oliviero, Roberta Ramonda, Martina Montagnana, Roberto Padoan

**Affiliations:** 1 Rheumatology Unit, Department of Medicine DIMED, University of Padua, Via Giustiniani, 2, 35128 Padua, Italy; 2 Laboratory Medicine Unit, Biomedical Sciences Department-DSB, University of Padua, Padua, Italy; 3 Laboratory Medicine Unit, AULSS 2 Marca Trevigiana, Treviso, Italy; 4 Department of Medicine DIMED, University of Padua, Padua, Italy

**Keywords:** ANCA, Vasculitis, Granulomatosis with polyangiitis, Microscopic polyangiitis, Inflammatory blood count ratios, Remission, Biomarker

## Abstract

Inflammatory ratios derived from routine complete blood counts, including the neutrophil-to-lymphocyte ratio (NLR), platelet-to-lymphocyte ratio (PLR), and monocyte-to-lymphocyte ratio (MLR), have been proposed as activity markers in ANCA-associated vasculitis (AAV), but their interpretation is limited by heterogeneous sampling and treatment-related confounding. We conducted a single-center cross-sectional study including adult patients with granulomatosis with polyangiitis (GPA) or microscopic polyangiitis (MPA). Patients were sampled during active disease (new-onset prior to induction or relapse prior to escalation) or during stringent glucocorticoid-free remission (BVASv3 = 0). Healthy controls (HCs) were included for secondary comparisons. Ratios were compared across groups and correlated with BVASv3 and c-reactive protein (CRP). Between-group differences (active vs. remission; remission vs. healthy controls) were additionally quantified using age- and sex-adjusted log-linear models (log-transformed outcomes), reported as ratios of geometric means. Discriminative performance for active disease versus remission was assessed using unadjusted ROC analyses and age- and sex-adjusted logistic regression models. Sensitivity analyses stratified remission by maintenance immunosuppression at sampling. The study included 99 AAV patients (28 active, 71 remission) and 258 HCs. NLR, PLR, and MLR were significantly higher in active disease than in remission (all *p* ≤ 0.004), and remained significantly higher after age/sex adjustment in log-linear models (geometric mean ratios [Active/Remission]: NLR 1.97, PLR 1.64, MLR 1.40). NLR and PLR correlated moderately with BVASv3 (ρ = 0.506 and 0.478, respectively; both *p* < 0.001) and CRP. Discriminative performance was strongest for NLR and PLR: in age- and sex-adjusted models, AUC increased from 0.664 (age/sex only) to 0.832 after adding NLR and 0.827 after adding PLR (whereas the AUC after adding MLR was 0.724). During glucocorticoid-free remission, ratios remained higher than in HCs (geometric mean ratios: NLR 1.55 [1.39–1.74], PLR 1.18 [1.08–1.29], MLR 1.40 [1.27–1.55]; all *p* < 0.001). Treatment stratification within remission showed lower lymphocyte counts and higher PLR in patients on maintenance immunosuppression, while NLR discrimination remained robust across strata. Routine blood count–derived inflammatory ratios, particularly NLR and PLR, discriminate active AAV from stringent glucocorticoid-free remission independent of age/sex, and remain persistently altered during remission. These inexpensive and widely available indices may complement clinical assessment, warranting prospective validation and evaluation of prognostic value.

**Supplementary Information:**

The online version contains supplementary material available at https://doi.org/10.1007/s10238-026-02236-x.

## Introduction

Anti-neutrophil cytoplasmic antibody (ANCA)-associated vasculitis (AAV) is a group of rare, severe inflammatory diseases of small- and medium-sized blood vessels, characterized by necrotizing vasculitis, extravascular granulomas, ANCA positivity, and multi-organ involvement. The main disease phenotypes include granulomatosis with polyangiitis (GPA), microscopic polyangiitis (MPA), and eosinophilic granulomatosis with polyangiitis (EGPA) [1]. 

AAV pathogenesis reflects a coordinated dysregulation of innate and adaptive immunity, involving neutrophils, monocytes, lymphocytes, autoantibodies (ANCA), and the complement system, ultimately leading to vascular inflammation and tissue damage [2]. Neutrophils play a central role as both ANCA targets and major effectors of tissue injury, while T cells contribute to ANCA production and amplification of inflammatory responses. Monocytes further sustain vascular inflammation through antigen presentation, cytokine release, and ANCA-dependent activation [3].

Despite this immunopathogenic complexity, disease activity is still primarily assessed using clinical tools such as the Birmingham Vasculitis Activity Score version 3 (BVASv3) [4], which may be insensitive to subtle changes. Similarly, commonly used laboratory biomarkers, including C-reactive protein (CRP), lack specificity, and ANCA titers have shown only modest accuracy in predicting relapse [5, 6]. Although immune profiling has proven useful in other autoimmune diseases [7, 8], reliable immune signatures reflecting AAV activity remain poorly defined.

Complete blood count–derived composite ratios, including the neutrophil-to-lymphocyte ratio (NLR), platelet-to-lymphocyte ratio (PLR), monocyte-to-lymphocyte ratio (MLR) have been proposed as accessible markers of activity and prognosis in AAV [9–13]. However, their clinical interpretation remains limited by heterogeneous sampling times, incomplete characterization of treatment exposure at time of sampling, reliance on in-sample cut-offs, and the intrinsic sensitivity of lymphocyte-based ratios to pharmacologic effects.

To address these limitations, the primary aim of this study was to evaluate composite inflammatory ratios across well-defined clinically activity states, comparing active AAV sampled prior to induction therapy or relapse-directed treatment escalation with a stringent glucocorticoid (GC)-free remission subgroup. We evaluated their ability to discriminate active disease from remission. As a secondary aim, we compared inflammatory ratios between patients in remission and healthy controls (HCs), to explore whether persistent differences may represent a biological signature associated with prior disease activity and/or ongoing maintenance therapy.

## Methods

### Study design

We conducted a cross-sectional study comparing hematological inflammatory ratios of patients with GPA and MPA in active disease, patients in clinical remission, and HCs. To minimize biological heterogeneity and to improve interpretation of hematological inflammatory ratios, EGPA was excluded because eosinophil-driven inflammation may substantially influence leukocyte-derived ratios.

We included adult patients (≥ 18 years) with GPA and MPA, followed at the Padua Vasculitis Center (Rheumatology Unit, Azienda Ospedale-Università degli Studi di Padova), diagnosed between June 2008 and September 2025 and classified according to 2012 Chapel Hill Consensus Conferences with 2012 update [1] or 2022 ACR/EULAR classification criteria [14, 15]. Sample collection and laboratory analysis were performed between January 2022 and September 2025. The study included both retrospectively and prospectively collected blood samples. For patients who had already undergone blood sampling during the study period and met eligibility criteria, laboratory results were retrieved retrospectively from electronic records. Additional eligible patients were enrolled prospectively, and sampled during routine clinical care throughout the study period. No complete blood count measurements were performed on frozen or stored samples.

Disease activity was assessed using the BVASv3. Active disease was defined as BVASv3 greater than 0 and remission as a BVASv3 score of 0 in the absence of vasculitis manifestations, according to current EULAR recommendations [16]. Relapse was defined as the recurrence of disease activity attributable to AAV (BVASv3 > 0) occurring after a period of stable clinical remission (BVASv3 = 0) documented at routine follow-up visits. To minimize treatment related confounding, a stringent remission subgroup was defined. Patients in remission were required to be free from systemic GCs and from immunosuppressive agents known to substantially cause marked perturbations in peripheral blood cell counts, particularly neutrophils, monocytes and platelet populations.

The study was conducted in accordance with the principles of the Declaration of Helsinki and received ethical approval from the Local Ethics Committee (CET-ACEV 6321/AO/25 May 25, 2025). Written informed consent was obtained from all participants.

### Population included

Eligible patients had positive MPO or PR3-ANCA. Exclusion criteria included negative ANCA serology at diagnosis, drug-induced vasculitis [17, 18] and conditions that may affect circulating blood cell profiles, such as ongoing infection, malignancy, hematologic disorders, other systemic autoimmune and rheumatological diseases, pregnancy and recent blood transfusion (within 3 months from the sample collection). Additionally, patients receiving treatments or procedures known to affect neutrophil and monocyte populations, including dialysis, plasmapheresis, intravenous immunoglobulins and selected immunosuppressive agents (cyclophosphamide, methotrexate [19], mycophenolate mofetil [20], colchicine, tacrolimus, cyclosporine and GCs [21, 22]) were also excluded. Maintenance treatment with rituximab (RTX) and azathioprine (AZA) was permitted, because they are less likely to induce marked neutrophil, monocyte and platelet shifts. For newly diagnosed patients, blood sampling was performed at first presentation before initiation of induction therapy. For relapsing patients, samples were obtained at relapse presentation before treatment escalation; patients were in documented remission at the preceding follow-up visit and were clinically stable prior to relapse onset.

HCs were recruited from healthy volunteers (without acute illness, relevant comorbidities, and leukocyte-modifying medications). Samples were processed under identical pre-analytical conditions and analyzed anonymously during the same study period and in the same laboratory.

### Data collection at diagnosis and at sampling

For each patient, demographic, clinical, laboratory and treatment data were collected at diagnosis and at the time of blood sampling.

Data collected at diagnosis included sex, age, dates of symptom onset and diagnosis, AAV subtype (MPA or GPA), clinical manifestations, ANCA status and specificity (MPO- or PR3-ANCA), CRP levels, and treatment history, including induction and maintenance regimens. Disease activity and severity were assessed using BVASv3 [4] and the Five Factor Score (FFS) [23].

At the time of sampling, collected data included age, disease duration, previous relapses, remission duration, clinical features, BVASv3, ANCA status and titer, CRP levels, duration and discontinuation date of previous GC or immunosuppressive therapies, current treatment, and organ damage assessed by the Vasculitis Damage Index (VDI) [24].

Complete blood counts were performed on fresh peripheral blood samples obtained as part of routine clinical care and analyzed at the time of collection by the institutional Laboratory Medicine Unit using the same standardized automated hematology analyzers. Hematological data were derived either retrospectively from electronic laboratory records or prospectively from samples collected during routine clinical visits, depending on the time of patient inclusion. Routine laboratory measurements included white blood cell (WBC), neutrophil, lymphocyte, monocyte and platelet count. Inflammatory ratios were calculated from complete blood counts as follows: the neutrophil-to-lymphocyte ratio (NLR), platelet-to-lymphocyte ratio (PLR), and monocyte-to-lymphocyte ratio (MLR), defined as the ratio of neutrophil, platelet, or monocyte counts to lymphocyte counts, respectively. Since the hematological variables required to calculate NLR, PLR, and MLR were retrieved from collected samples, no missing data were present for these hematological variables.

For HCs, age, sex, and complete blood count parameters retrieved from retrospectively from electronic records were collected.

### Statistical analysis

Categorical variables are reported as numbers and percentages, whereas continuous variables as median and interquartile ranges (IQRs). A limited number of missing values were present for some secondary variables (e.g. CRP), and analyses involving those variables were performed on available cases only. Group comparisons were performed using non-parametric tests due to non-normal data distribution (Shapiro–Wilk test). Chi-square tests or Fisher’s exact tests were used for categorical variables, while Mann–Whitney U test (two groups) or Kruskal–Wallis test (≥ 3 groups) was applied for continuous variables, as appropriate. Correlations were assessed using Spearman’s rank correlation coefficient (ρ).

Given the imbalance in age between HCs and patients, comparisons were repeated using age- and sex-adjusted models. Between-group (active vs. remission and remission vs. HCs) differences in inflammatory ratios were estimated using log-linear regression models with log-transformed ratios as outcomes (to account for right-skewness), including group as the main predictor and adjusting for age and sex; adjusted effects are reported as ratios of geometric means with 95% confidence intervals. To evaluate discriminative ability for disease activity (active vs. remission), we used receiver operating characteristic (ROC) analyses. The area under the curve (AUC) was the primary metric used to compare discriminative ability across indices, considering the age- and sex-adjusted model alone and after addition of each index. To account for demographic differences, age- and sex-adjusted logistic regression models were fitted including each inflammatory index as predictor (log-transformed to account for right-skewness). Effect estimates are presented as odds ratios (ORs) per 1 standard deviation (SD) increase in the log-transformed index to provide a standardized, with 95% confidence intervals (CIs). To improve clinical interpretability, we additionally report ORs per doubling of each ratio using log2-transformed predictors.

As sensitivity analyses to address treatment-related lymphocyte variation, remission samples were stratified by immunosuppression at time of sampling (on vs. off), and age- and sex-adjusted logistic models were repeated within each stratum.

Unless stated, a two-sided p-value < 0.05 was considered statistically significant. All analyses and figures were performed using Jamovi (version 2.4.11), IBM SPSS Statistics (version 26) and GraphPad Prism (version 10.1.1).

## Results

### Subject characteristics

The patient selection process is summarized in Supplementary Fig. 1. Briefly, 271 patients followed at the Padua Vasculitis Center were screened for eligibility. Of 112 patients considered eligible for sampling, 8 were excluded after sampling because of newly identified exclusion criteria, and 5 eligible patients were not sampled owing to logistical reasons or refusal to participate. The final study population included 99 AAV patients, of whom 28 were sampled during active disease and 71 during clinical remission, together with 258 healthy controls, accounting for a total of 357 participants.

Of the active disease samples, 23/28 (82%) were obtained at diagnosis and 5/28 (18%) during relapse, after a median of 155 [84–162] months of stable remission, with duration calculated from the date of first remission (BVASv3 = 0) achievement to the relapse sample date.

Overall, 75 patients were classified as GPA and 24 patients as MPA. The distribution of AAV subtypes was similar across activity states: during active disease, 21/75 (28.0%) of GPA patients and 7/24 (29.2%) of MPA patients were sampled. PR3-ANCA specificity at diagnosis was also comparable between groups, being present in 19/28 (67.9%) patients sampled during active disease and 51/71 (71.8%) of those sampled in remission.

Comprehensive demographic and clinical characteristics at diagnosis and at the time of sampling are reported in Supplementary Table 1 and Table 1.

HCs were younger than patients (median age 47 [31–58] years vs. 60 [51–70] years, *p* < 0.001), and the proportion of females differed between groups (27.9% [72/258] in HCs vs. 50.5% [50/99] in AAV patients, *p* < 0.001).

Among patients sampled in remission, the median disease duration at the time of sampling was 84.0 [46.5–153.5] months, and the median duration of remission was 49 [18.5–95.5] months. The median time since GC discontinuation was 42.0 (13.5–71.0) months. In the subgroup not receiving any maintenance immunosuppressive therapy (29/71, 40.8%), the median time since immunosuppression withdrawal was 21.0 (IQR 10.0–64.5) months. Overall, 38.0% of patients in remission had a prior history of relapse.

**Table 1:** Demographic and clinical characteristics at sampling of AAV patients, according to clinical status

		Active (*n* = 28)	Remission (*n* = 71)
Demographics	Male, *n* (%)	19/28 (67.9)	30/71 (42.3)
	Age, years	61.0 [51.8–77.3]	60.0 [49.0–68.0]
	Disease duration, months	0 [0–0]	84.0 [46.5-153.5]
**Laboratory data**	ANCA positivity, n (%)	28/28 (100)	33/71 (46.5)
	CRP, mg/L	41.4 [14.8-144.6]	2.3 [1.2-3.0]
**Disease course**	BVASv3	16.5 [12.0–21]	0 [0–0]
	VDI	-	4 [2–5]
	Remission duration, months	-	49 [18.5–95.5]
	Time off GC, months	-	42 [13.5–71.0]
	Time off IS, months	-	21 [10.0-64.5]
	Previous relapse, n (%)	2/28 (7.1)	27/71 (38.0)
**Treatment**	IS, n (%)	4/28 (14.3)	42/71 (59.2)
	Rituximab, n (%)	1/28 (3.6)	38/71 (53.6)
	Azathioprine, n (%)	3/28 (10.7)	4/71 (5.6)
	No treatment, n (%)	24/28 (85.7)	29/71 (40.8)

Data are expressed as median and interquartile range (IQR). ANCA, Anti-neutrophil cytoplasmic antibodies; BVASv3, Birmingham Vasculitis Activity Score version 3; CRP, c-reactive protein; GC, glucocorticoids; IS, immunosuppressant; VDI, vasculitis damage index

### Hematologic parameters and composite ratios across activity states

Compared with patients in remission and HCs, patients with active AAV had significantly higher total WBC counts (active: 8.32 [6.88–12.55] vs. remission: 7.03 [5.97–8.30] vs. HCs: 5.93 [5.22–6.57] ×10⁹/L; *p* < 0.001). This pattern was driven by higher neutrophil counts (6.20 [5.14–10.34] vs. 4.50 [3.48–5.53] vs. 3.22 [2.70–3.75] ×10⁹/L; *p* < 0.001), higher monocyte counts (0.74 [0.42–1.17] vs. 0.61 [0.46–0.83] vs. 0.51 [0.43–0.62] ×10⁹/L; *p* < 0.001), and higher platelet counts (314 [242–376] vs. 257 [222–290] vs. 237 [222–290] ×10⁹/L; *p* < 0.001), alongside lower lymphocyte counts (1.42 [1.10–1.68] vs. 1.66 [1.30–2.21] vs. 1.89 [1.62–2.35] ×10⁹/L; *p* < 0.001). Pairwise comparisons showed that patients with active disease differed significantly from patients in remission and from HCs for all parameters, except for monocyte counts, which were comparable between active disease and remission (*p* = 0.403). Patients in remission also differed significantly from HCs for WBC, neutrophils, monocytes and lymphocytes (*p* = 0.001 for each), whereas the difference in platelet count was smaller (*p* = 0.019). Comprehensive data are reported in Table 2.

**Fig. 1:**
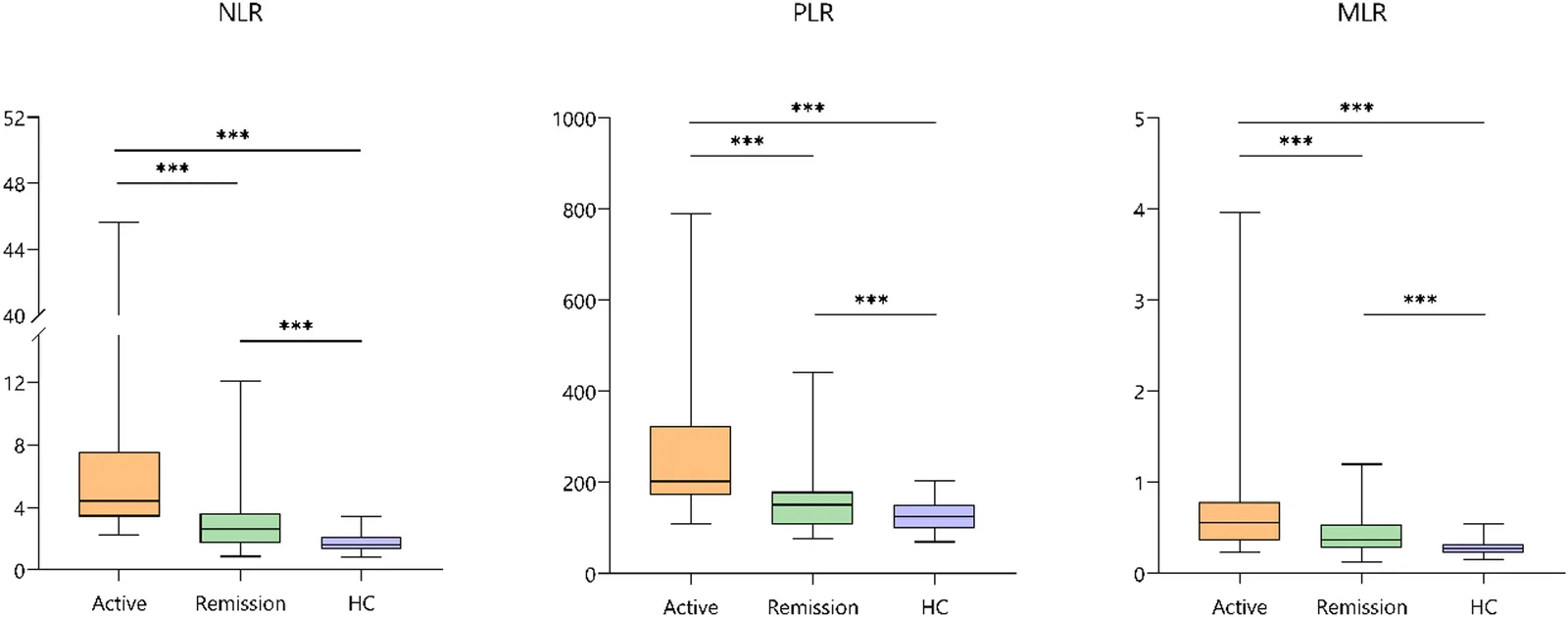
Inflammatory ratios in active AAV, remission, and HCs. Box plots represent median and interquartile range; whiskers indicate the range. P values refer to between-group comparisons using Mann–Whitney U test. Asterisks indicate statistically significant differences (*p* < 0.001)

The NLR, PLR and MLR were markedly higher in the active phase, and lower in remission (NLR: 4.40 [3.45–7.53] vs. 2.60 [1.76–3.55], *p* < 0.001; PLR: 202.9 [176.2-292.5] vs. 151.2 [109.8-179.5], *p* < 0.001; MLR: 0.56 [0.40–0.78] vs. 0.37 [0.27–0.53], *p* = 0.004). Patients in remission exhibited also higher NLR, PLR and MLR compared to HCs (*p* < 0.001 for all parameters). Data on laboratory parameters are reported in Table 2; Fig. 1 and Supplementary Table 2.

**Table 2:** Absolute leukocyte subsets, platelet counts, and composite inflammatory ratios in AAV and HCs

	Active (*n* = 28)	Remission (*n* = 71)	HCs (*n* = 258)	*P* value active vs. remission^1^	*P* value remission vs. HCs^1^	*P* value active vs. HCs^1^	*P* value^2^
White blood cells, x 10^9/L	8.32 [6.88–12.55]	7.03 [5.97–8.30]	5.93 [5.22–6.57]	0.003	< 0.001	< 0.001	< 0.001
Neutrophils, x 10^9/L	6.20 [5.14–10.34]	4.50 [3.48–5.53]	3.22 [2.70–3.75]	**< **0.001	**< **0.001	**< **0.001	**< **0.001
Lymphocytes, x 10^9/L	1.42 [1.10–1.68]	1.66 [1.30–2.21]	1.89 [1.62–2.35]	0.021	0.001	**< **0.001	**< **0.001
Monocytes, x 10^9/L	0.74 [0.42–1.17]	0.61 [0.46–0.83]	0.51 [0.43–0.62]	0.403	**< **0.001	0.022	**< **0.001
Platelets, x 10^9/L	314 [242–376]	257 [222–290]	237 [222–290]	0.002	0.019	**< **0.001	**< **0.001
NLR	4.40 [3.45–7.53]	2.60 [1.76–3.55]	1.63 [1.32–2.11]	**< **0.001	**< **0.001	**< **0.001	**< **0.001
PLR	202.9 [176.2-292.5]	151.2 [109.8-179.5]	124.6 [99.0-150.6]	**< **0.001	**< **0.001	**< **0.001	**< **0.001
MLR	0.56 [0.40–0.78]	0.37 [0.27–0.53]	0.27 [0.22–0.32]	0.004	**< **0.001	**< **0.001	**< **0.001

Data are expressed as median and interquartile range (IQR). ^1^Mann–Whitney U test, ^2^ Kruskal–Wallis test. MLR, Monocytes to lymphocytes ratio; NLR, Neutrophils to lymphocytes ratio; PLR, Platelets to lymphocytes ratio

To provide an interpretable adjusted effect size, we additionally fitted age- and sex-adjusted log-linear models (log-transformed ratios). In these models ratios remained higher in active disease than in remission, with adjusted geometric mean ratios (Active/Remission) of 1.97 (95% CI 1.52–2.54, *p* < 0.001) for NLR, 1.64 (95% CI 1.36–1.99, *p* < 0.001) for PLR and 1.40 (95% CI 1.09–1.79, *p* = 0.008) for MLR. In the secondary analysis comparing remission with HCs, age- and sex-adjusted log-linear models confirmed that NLR, PLR and MLR remained higher in remission than in HCs, with ratios of geometric means (Remission/HC) of 1.55 (95%CI 1.39–1.74, *p* < 0.001) for NLR, 1.18 (95%CI 1.08–1.29, *p* < 0.001) for PLR and 1.40 (95%CI 1.27–1.55, *p* < 0.001) for MLR.

Within the remission subgroup, 42 patients were receiving maintenance treatment at the time of sampling (RTX *n* = 38, AZA *n* = 4), while 29 were off immunosuppression. Compared with those off treatment, patients on maintenance therapy had lower lymphocyte counts (1.48 [1.08–1.97] vs. 2.06 [1.54–2.32] ×10⁹/L; *p* = 0.009) and higher PLR (162 [125–198] vs. 118 [101–157]; *p* = 0.006), whereas NLR was similar between groups. Within the remission subgroup, no additional clinically relevant differences were observed in inflammatory ratio according to demographic, serological and disease characteristics (Supplementary Table 3).

### Correlations with disease activity, inflammation and disease history

**Fig. 2:**
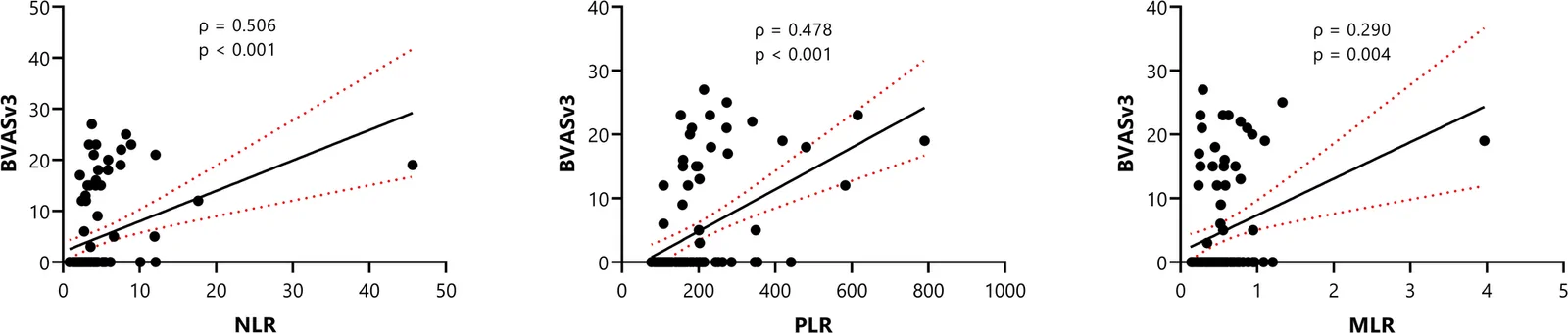
Correlation between inflammatory ratio and disease activity. Correlation between disease activity, assessed by the Birmingham Vasculitis Activity Score version 3 (BVASv3), and inflammatory ratios in patients with AAV. Neutrophil-to-lymphocyte ratio (NLR, left panel), platelet-to-lymphocyte ratio (PLR, central panel) and monocyte-to-lymphocyte ratio (MLR, righ panel) show significant positive correlations with BVASv3 (Spearman’s ρ = 0.506 and 0.478, for NLR and MLR respectively; both *p* < 0.001; Spearman’s ρ = 0.290, *p* = 0.004 for MLR). Solid black lines represent linear regression fits, while dotted lines indicate 95% confidence intervals

In the overall AAV cohort (*n* = 99), NLR and PLR showed moderate correlation with BVASv3 at the time of sampling (ρ = 0.506 and 0.478, respectively; both *p* < 0.001), whereas MLR showed a weaker correlation (ρ = 0.289, *p* = 0.004), Table 3. Consistently, in linear regression analyses, BVASv3 increased with higher NLR (slope = 0.595, 95%CI 0.296–0.894), PLR (slope = 0.0328, 95%CI 0.020–0.045) and MLR (slope = 0.290, 95%CI 2.169–9.265), Fig. 2.

NLR and PLR also correlated with CRP levels (ρ = 0.344, *p* < 0.001 and ρ = 0.324, *p* = 0.001), whereas no significant correlation was observed for MLR. When analyses were restricted to active samples only (*n* = 28), correlations between lymphocyte-based ratios and BVASv3 remained directionally consistent but did not reach statistical significance, likely reflecting limited power.

With respect to disease history, in the overall AAV cohort (*n* = 99) ratios were inversely associated with disease duration at time of sampling (NLR ρ= −0.302, PLR ρ= −0.327, MLR ρ = −0.221). In the remission subgroup with damage assessment available (*n* = 71), NLR and MLR correlated with VDI (ρ = 0.387, *p* < 0.001; ρ = 0.296, *p* = 0.012). No significant associations were observed between any index and a prior history of relapse.

In sensitivity correlation analyses adjusted for absolute lymphocyte count and CRP or BVASv3, NLR and PLR remained the ratios most consistently associated with BVASv3. In the remission subgroup, NLR, PLR and MLR did not show significant correlations with disease duration at the time of sampling or with time since GC or immunosuppressive therapy discontinuation (Supplementary Table 4).

**Table 3:** Spearman’s correlation coefficients (unadjusted and adjusted) between blood counts indices and disease activity in AAV

A: Unadjusted correlations (Spearman’s ρ)				
Index	BVASv3 (*n* = 99)		CRP (*n* = 94)	
	ρ	*P* value	ρ	*P* value
NLR	**0.506**	< 0.001	**0.344**	< 0.001
PLR	**0.478**	< 0.001	**0.324**	0.001
MLR	**0.289**	0.004	0.151	0.147
Lymphocytes (^10⁹/L)	**-0.253**	0.011	-0.065	0.531

B: Adjusted correlations (sensitivity analyses)				
Index	BVASv3*		CRP†	
	Adjusted ρ	*P* value	Adjusted ρ	*P* value
NLR	**0.303**	0.003	0.110	0.295
PLR	**0.282**	0.007	0.159	0.129
MLR	0.106	0.314	0.031	0.768

* Adjusted for lymphocyte count and CRP

† Adjusted for lymphocyte count and BVASv3

Panel A shows Spearman’s rho (ρ) for correlations of indices with BVASv3 (*n* = 99) and CRP (*n* = 94). Panel B shows partial correlations adjusted for absolute lymphocyte count and CRP (for BVASv3) or absolute lymphocyte count and BVASV3 (for CRP). Values highlighted in bold represent statistically significant p values (*p* < 0.05).

### ROC curves analyses and model-based discrimination

**Fig. 3:**
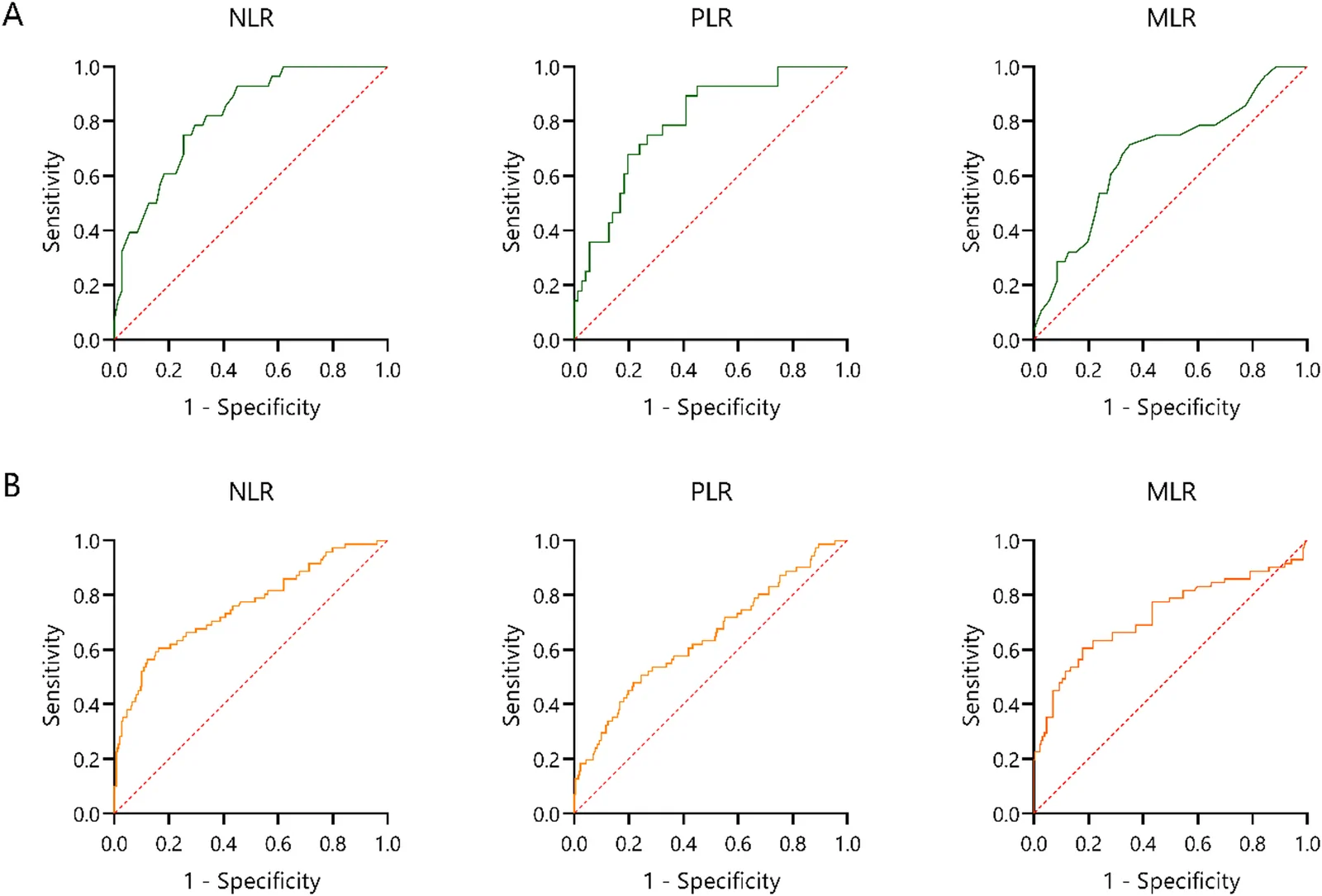
Discriminative performance of inflammatory ratios for disease activity in ANCA-associated vasculitis and healthy controls. Receiver operating characteristic (ROC) curves showing the ability of neutrophil-to-lymphocyte ratio (NLR), platelet-to-lymphocyte ratio (PLR), and monocyte-to-lymphocyte ratio (MLR) to discriminate disease activity in ANCA-associated vasculitis. **(A)** ROC curves for discrimination between active disease and clinical remission. For NLR, the area under the curve was 0.818, with sensitivity and specificity of 78.57% and 71.83%, respectively. For PLR, the area under the curve was 0.795, with 89.29% sensitivity and 59.15% specificity. For MLR, the area under the curve was 0.684, with 71.43% sensitivity and 67.61% specificity. **(B)** ROC curves for discrimination between patients in remission and HCs. The diagonal dashed line represents the reference line of no discrimination. For NLR, the area under the curve was 0.757, with sensitivity and specificity of 56.34% and 87.98%, respectively. For PLR, the area under the curve was 0.644, with 50.70% sensitivity and 75.58% specificity. For MLR, the area under the curve was 0.726, with 60.56% sensitivity and 82.17% specificity

Unadjusted ROC analyses were performed to assess the ability of NLR, PLR and MLR to discriminate active disease from remission, using BVASv3 as reference. All three ratios significantly differentiated active disease from remission (NLR and PLR: *p* < 0.001; MLR: *p* = 0.004), with NLR showing the highest discriminative ability (AUC 0.818, 95%CI: 0.732–0.903), followed by PLR (AUC 0.795, 95%CI: 0.701–0.889) and MLR (AUC 0.684, 95%CI: 0.565–0.803) (Fig. 3; Table 4). The Youden-derived optimal cut-off for NLR was 3.35, corresponding to 78.6% sensitivity and 71.8% specificity (Table 4).

**Table 4:** Unadjusted discrimination performance of composite inflammatory ratios

Parameter	AUC (95% CI)	*P* value	Youden Index	Cut-off value	Sensitivity (%)	Specificity (%)
**AAV patients**						
NLR	0.818 (0.732–0.903)	< 0.001	0.504	3.35	78.57	71.83
PLR	0.795 (0.701–0.889)	< 0.001	0.484	158.3	89.29	59.15
MLR	0.684 (0.565–0.803)	0.004	0.390	0.44	71.43	67.61
**Patients in remission and HCs**						
NLR	0.757 (0.688–0.825)	< 0.001	0.443	2.45	56.34	87.98
PLR	0.644 (0.567–0.720)	< 0.001	0.263	151.0	50.70	75.58
MLR	0.726 (0.648–0.803)	< 0.001	0.427	0.34	60.56	82.17

The optimal cut-off was extrapolated via calculating the area under the receiver operator characteristic (AUROC) curve and determined using the Youden index, defined as the maximum value of sensitivity + specificity – 1. MLR, Monocytes to lymphocytes ratio; NLR, Neutrophils to lymphocytes ratio; PLR, Platelets to lymphocytes ratio

To account for demographic differences, we also quantified discrimination using age- and sex-adjusted logistic regression models. In the primary comparison (active vs. remission), NLR and PLR showed the strongest associations with active disease (OR per 1 SD increase in log-ratio: 4.27 [95% CI 2.05–8.88] and 3.51 [1.86–6.64], respectively; both *p* < 0.001), whereas MLR showed a more modest association (OR 1.97 [1.16–3.35], *p* = 0.013). For clinical interpretability, effect estimates are also reported per doubling of each ratio (log2 scale): 4.83 (95% CI 2.18–10.69) for NLR, 6.61 (2.54–17.21) for PLR, and 2.30 (1.20–4.43) for MLR. In discrimination analyses, the AUC of the model including age and sex alone was 0.664 and increased to 0.832 after adding NLR and to 0.827 after adding PLR, whereas the corresponding AUC after adding MLR was 0.724. In sensitivity analyses repeating the age- and sex-adjusted logistic models within remission strata, NLR remained a strong discriminator of active disease both among patients off immunosuppression (AUC 0.840) and on immunosuppression (AUC 0.844), and PLR also remained discriminative (AUC 0.873 and 0.801, respectively) (Supplementary Table 5).

In secondary unadjusted ROC analyses comparing remission vs. HCs, NLR again showed the highest discriminative ability (AUC 0.757, 95%CI: 0.688–0.825, *p* < 0.001), followed by MLR (AUC 0.726, 95%CI: 0.648–0.803, *p* < 0.001) and PLR (AUC 0.644, 95%CI: 0.567–0.720, *p* < 0.001) (Fig. 3; Table 4). The Youden-derived optimal cut-off for NLR was 2.45, with a sensitivity of 56.3%, and a specificity of 88.0% (Table 4). The AUC of the model including age and sex alone was 0.769 and increased to 0.850 after adding NLR and to 0.821 after adding MLR, whereas the corresponding AUC after adding PLR was 0.798.

## Discussion

In this single-center cross-sectional study, we investigated routine blood count–derived inflammatory ratios across clinically defined activity states in AAV. We found that NLR, PLR, and MLR were significantly elevated during active disease compared with both clinical remission and HCs, and correlated with established clinical and laboratory measures of activity, such as BVASv3 and CRP. Among these ratios, NLR demonstrated the strongest discriminative ability for active disease versus remission (unadjusted AUC 0.818, with similar performance in age/sex-adjusted models), with consistent performance when stratified by maintenance immunosuppression status. Notably, all three ratios remained significantly elevated during sustained GC-free clinical remission compared with healthy controls, despite prolonged immunosuppression withdrawal in a substantial proportion of patients.

NLR, PLR, and MLR reflect the systemic balance between innate and adaptive immune responses and have been associated with disease activity across several autoimmune and inflammatory conditions, including rheumatoid arthritis [25], polymyalgia rheumatica [26], Takayasu’s arteritis [27] and systemic lupus erythematosus [28, 29]. The elevation of these ratios in active AAV aligns with the central role of innate immune activation in disease pathogenesis. Neutrophils serve as both primary ANCA targets and key effectors of vascular injury through release of reactive oxygen species, proteolytic enzymes, and neutrophil extracellular traps [2]. Monocytes contribute to sustained inflammation through ANCA-mediated activation, antigen presentation, and proinflammatory cytokine production. Concurrently, the relative lymphopenia observed during active disease likely reflects recruitment of T cells, particularly CD4 + subsets, from peripheral circulation to sites of active inflammation [30, 31]. The resulting neutrophilia, monocytosis, thrombocytosis, and relative lymphopenia collectively drive higher composite ratios during active disease.

Our findings confirm and extend previous observations regarding the association between inflammatory ratios and AAV disease activity. The consistency of these findings across treatment-stratified sensitivity analyses strengthens confidence in their validity and suggests these ratios capture biologically relevant immune activation independent of pharmacologic effects. Elevated NLR has been previously linked to severe disease activity, increased relapse risk, and adverse outcomes including severe infections following induction therapy [13, 32, 33]. Similarly, increased platelet counts and PLR have been associated with renal involvement and severe disease [9], and higher NLR, PLR, and MLR have been reported in patients progressing to end-stage renal disease [34]. However, inconsistencies exist in the literature, with some earlier studies reporting conflicting results regarding the association between NLR and BVASv3 [35, 36].

Previous studies investigating inflammatory ratios in AAV have been constrained by several important methodological limitations that restrict their clinical interpretability. First, most reports have relied on in-sample, data-driven cut-offs, often using tertile-based stratification without external validation [12, 13], limiting generalizability and risking overfitting. Second, many studies have combined heterogeneous AAV subtypes, including EGPA [10, 12, 13, 34, 37], in which eosinophil-driven inflammation can substantially confound neutrophil-based ratios. Third, treatment-related confounding has been inadequately addressed in most prior work. GCs profoundly affect lymphocyte counts through inhibition of apoptosis and redistribution [21, 22], yet many studies have included remission samples obtained during ongoing GC therapy without stratification or adjustment. Similarly, agents such as cyclophosphamide, methotrexate, and mycophenolate substantially perturb neutrophil and monocyte populations [19, 20], but treatment exposure at sampling is often incompletely characterized [10, 37]. Fourth, control group selection and comparability have been inconsistent or absent across studies. Some reports lack healthy control comparisons entirely, precluding assessment of whether remission values normalize or remain altered [34]. Finally, most prior studies have been conducted exclusively in Asian or Middle Eastern populations [10, 12, 13, 37, 38], where genetic backgrounds and disease phenotypes may differ from Caucasian cohorts, limiting the generalizability of findings to European and North American populations.

A key and clinically relevant novel finding of our study is that inflammatory ratios remained significantly elevated in patients with sustained clinical GC-free remission compared with healthy controls, including among the 40.8% of patients with complete withdrawal of maintenance immunosuppression. Associations with disease duration and time since treatment discontinuation were generally weak, arguing against a purely time-dependent recovery effect. Several interpretations of this finding merit consideration. Persistent elevations may reflect subclinical immune dysregulation or undetected subclinical disease activity despite clinically-defined remission, consistent with immune profiling studies showing residual abnormalities in the absence of symptoms [7, 39]. They could also represent residual immune “scarring” from prior activity or, in part, treatment-related effects; however, treatment-stratified analyses suggested that NLR remained elevated even among patients off immunosuppression. The clinical significance of these persistent alterations warrants prospective investigation.

The main strength of this study is the inclusion of a biologically-defined contrast by comparing active disease sampled prior to treatment initiation or before relapse-directed treatment escalation with a stringent remission cohort in documented clinical remission. Critically, our remission definition explicitly excluded systemic GCs and immunosuppressive agents, and we addressed potential treatment-related lymphocyte variation through treatment-stratified sensitivity analyses. This approach minimizes pharmacologic confounding and strengthens internal validity. However, it limits generalizability to the broader population of patients in remission who remain on glucocorticoids and/or combination immunosuppression. Finally, rather than relying primarily on data-driven thresholds, we report continuous associations with clinical outcomes using age- and sex-adjusted regression models, presenting cut-offs as exploratory and hypothesis-generating rather than as validated diagnostic thresholds.

This study has inherent limitations. Firstly, its single-center design and relatively modest sample size may limit generalizability and statistical power, particularly in subgroup analyses. Second, the cross-sectional design precludes assessment of intra-individual changes over time and limits causal inference regarding the relationship between ratios and disease activity. Moreover, residual effects of prior treatments cannot be entirely ruled out, particularly given the prolonged half-lives of some immunosuppressive agents and potential for long-lasting effects on immune cell populations. Comparisons between patients in remission and HCs should also be interpreted with caution and primarily viewed as exploratory evidence supporting persistent biological differences during remission, as controls were younger than patients with AAV and the study was not designed as an age-matched comparison. Although age- and sex-adjusted analyses were performed, residual confounding related to demographic differences may persist. Moreover, other possibilities for persistent elevation of indices during remission may also contribute to the observed differences from HCs. Finally, our study did not evaluate the predictive value of these ratios for long-term outcomes such as relapse, organ damage progression, or mortality, which represents an important direction for future research.

## Conclusions

In this single-center cross-sectional study, we showed that hematological inflammatory ratios derived from routine complete blood counts may complement existing clinical assessment tools in AAV by capturing aspects of immune activation not fully reflected in clinical disease activity scores. Our findings suggested that these readily available, standardized, low-cost biomarkers make them attractive candidates for integration into routine monitoring protocols, although their nonspecific nature requires cautious interpretation. Future prospective studies will be crucial to further define their role across different treatment settings and to explore their prognostic relevance.

## Supplementary Information

Below is the link to the electronic supplementary material.

## Data Availability

The data that support the findings of this study are available from the corresponding author upon reasonable request.
