# Supplementary material for: Inflammatory blood count ratios discriminate disease activity and remain persistently elevated during glucocorticoid-free remission in ANCA-associated vasculitis
Source: Clin Exp Med. 2026 Jun 22;26(1):318. doi: 10.1007/s10238-026-02236-x (PMC13538257; doi:10.1007/s10238-026-02236-x)
Supplement: Supplementary file 1 [file 10238_2026_2236_MOESM1_ESM.docx]

**Supplementary Table 1.** Demographic and clinical characteristics at diagnosis of AAV patients

|  |  | AAV patients (n = 99) |
| --- | --- | --- |
| Demographics | Male, n (%) | 49 (49.5) |
|  | Age at diagnosis, years | 51 [44-64] |
|  | GPA, n (%) | 72 (72.7) |
|  | MPA, n (%) | 24 (24.2) |
| Severity of vasculitis | FFS 0, n (%)  FFS 1, n (%)  FFS 2, n (%)  FFS 3, n (%) | 43 (43.4)  17 (17.3)  37 (37.4)  2 (2.0) |
|  | FFS1996 > 1, n (%) | 57 (57.6) |
|  | BVASv3 | 17 [12-21] |
| Organ involvement | General manifestations, n (%) | 77 (77.8) |
|  | Mucous membranes, n (%) | 26 (26.3) |
|  | Cutaneous, n (%) | 29 (29.3) |
|  | ENT, n (%) | 13 (46.4) |
|  | Chest, n (%) | 55 (55.5) |
|  | Cardiovascular, n (%) | 3 (3.0) |
|  | Abdominal, n (%) | 5 (5.0) |
|  | Renal, n (%) | 73 (73.7) |
|  | Nervous system, n (%) | 21 (21.2) |
| ANCA type | ANCA-PR3, n (%) | 70 (70.7) |
|  | ANCA-MPO, n (%) | 9 (29.3) |

*Data are expressed as median) and interquartile range (IQR).*

*AAV, ANCA associated vasculitis; ANCA, Anti-neutrophil cytoplasmic antibodies; BVASv3, Birmingham Vasculitis Activity Score version 3; FFS, Five Factor Score; ENT, ear, nose and throat; GPA, granulomatosis with polyangiitis; MPA, microscopic polyangiitis; MPO, myeloperoxidase; PR3, proteinase 3.*

**Supplementary Table 2.** Differences in inflammatory ratio according to sex and age in HCs

|  | **Inflammatory indices** | |  |
| --- | --- | --- | --- |
| **NLR** | **Variable present** | **Variable absent** | **P value^1^** |
| Female | 1.60 (1.27-2.08) | 1.64 (1.40-2.13) | 0.363 |
| Age > 60 y | 1.64 (1.45-2.20) | 1.62 (1.27-2.10) | 0.202 |
| **PLR** | **Variable present** | **Variable absent** | **P value^1^** |
| Female | 123.0 (102.6-148.6) | 124.9 (97.7-150.7) | 0.697 |
| Age > 60 y | 133.3 (109.8-152.2) | 121.7 (96.7-149.7) | 0.139 |
| **MLR** | **Variable present** | **Variable absent** | **P value^1^** |
| Female | 0.23 (0.20-0.29) | 0.27 (0.23-0.33) | **< 0.001** |
| Age > 60 y | 0.29 (0.25-0.34) | 0.26 (0.22-0.31) | **0.017** |

*Data are expressed as median and interquartile range (IQR). ^1^ Mann–Whitney U test. MLR, Monocytes to lymphocytes ratio; NLR, Neutrophils to lymphocytes ratio; PLR, Platelets to lymphocytes ratio.*

**Supplementary Table 3.** Inflammatory ratio by demographic, serological, and disease characteristics at sampling of AAV patients in remission

| **AAV patients in remission** | **Inflammatory indices** | |  |
| --- | --- | --- | --- |
| **NLR** | **Variable present** | **Variable absent** | **P value^1^** |
| Female | 2.55 (1.69-3.22) | 2.94 (1.83-4.31) | 0.234 |
| Age > 60 y | 2.60 (2.19-3.98) | 2.26 (1.15-3.42) | 0.310 |
| ANCA subtype at diagnosis | 2.66 (1.93-3.77) | 2.33 (1.67-3.35) | 0.447 |
| ANCA positivity at sampling | 2.96 (2.35-3.56) | 2.10 (1.57-3.46) | 0.152 |
| Previous relapse | 2.91 (1.75-4.09) | 2.55 (1.76-3.34) | 0.809 |
| Remission duration > 4 y | 2.59 (1.84-3.34) | 2.59 (1.58-4.14) | 0.977 |
| IS at sampling | 2.60 (1.96-3.89) | 2.59 (1.69-3.43) | 0.588 |
| RTX at sampling | 2.60 (1.93-4.01) | 2.59 (1.75-3.43) | 0.667 |
| **PLR** | **Variable present** | **Variable absent** | **P value^1^** |
| Female | 153.4 (117.5-177.1) | 143.1 (99.3-179.7) | 0.435 |
| Age > 60 y | 139.9 (111.4-177.1) | 158.7 (110.5-183.1) | 0.573 |
| ANCA subtype at diagnosis | 151.1 (112.6-178.4) | 146.2 (107.8-195.3) | 0.873 |
| ANCA positivity at sampling | 156.7 (122.8-192.4) | 131.9 (104.7-177.1) | 0.116 |
| Previous relapse | 156.8 (125.8-189.5) | 144.5 (107.8-177.6) | 0.397 |
| Remission duration > 4 y | 134.5 (101.2-167.2) | 156.8 (122.1-184.6) | 0.093 |
| IS at sampling | 162.2 (124.8-197.9) | 117.9 (100.8-156.8) | **0.006** |
| RTX at sampling | 163.8 (133.4-197.9) | 121.7 (101.4-156.8) | **0.006** |
| **MLR** | **Variable present** | **Variable absent** | **P value^1^** |
| Female | 0.34 (0.27-0.44) | 0.40 (0.27-0.60) | 0.299 |
| Age > 60 y | 0.41 (0.29-0.58) | 0.34 (0.25-0.46) | **0.045** |
| ANCA subtype at diagnosis | 0.39 (0.28-0.54) | 0.31 (0.21-0.48) | 0.161 |
| ANCA positivity at sampling | 0.40 (0.33-0.56) | 0.36 (0.26-0.50) | 0.277 |
| Previous relapse | 0.39 (0.28-0.56) | 0.36 (0.27-0.51) | 0.469 |
| Remission duration > 4 y | 0.39 (0.27-0.47) | 0.37 (0.27-0.58) | 0.676 |
| IS at sampling | 0.40 (0.29-0.60) | 0.33 (0.25-0.42) | 0.055 |
| RTX at sampling | 0.39 (0.28-0.61) | 0.34 (0.27-0.42) | 0.009 |

*Data are expressed as median and interquartile range (IQR). ^1^ Mann–Whitney U test. ANCA, Anti-neutrophil cytoplasmic antibodies; IS, immunosuppressant; MLR, Monocytes to lymphocytes ratio; NLR, Neutrophils to lymphocytes ratio; PLR, Platelets to lymphocytes ratio; RTX, rituximab.*

**Supplementary Table 4**. Spearman correlation analysis of disease history and laboratory parameters in AAV patients in remission

|  | **Remission disease duration** | | **Time since GC discontinuation** | | **Time since IS discontinuation (n = 29)** | |
| --- | --- | --- | --- | --- | --- | --- |
|  | **ρ** | **P value** | **ρ** | **P value** | **ρ** | **P value** |
| **PLR** | - 0.199 | 0.097 | - 0.094 | 0.433 | -0.125 | 0.475 |
| **NLR** | 0.025 | 0.837 | 0.071 | 0.553 | -0.150 | 0.389 |
| **MLR** | - 0.064 | 0.593 | -0.066 | 0.584 | -0.170 | 0.329 |
| **Lymphocytes** | 0.136 | 0.256 | 0.029 | 0.808 | 0.138 | 0.429 |

*GC, glucocorticoids; IS, immunosuppressant; MLR, Monocytes to lymphocytes ratio; NLR, Neutrophils to lymphocytes ratio; PLR, Platelets to lymphocytes ratio; ρ: Spearman’s rho.*
